# Regulation of Skp2 Expression and Activity and Its Role in Cancer Progression

**DOI:** 10.1100/tsw.2010.89

**Published:** 2010-06-01

**Authors:** Chia-Hsin Chan, Szu-Wei Lee, Jing Wang, Hui-Kuan Lin

**Affiliations:** ^1^ Department of Molecular and Cellular Oncology, University of Texas, M.D. Anderson Cancer Center, Houston, TX, USA; ^2^ University of Texas, Graduate School of Biomedical Sciences, Houston, TX, USA

**Keywords:** Skp2, Akt, p27, SCF complex, phosphorylation, neddylation, cell migration, cancer

## Abstract

The regulation of cell cycle entry is critical for cell proliferation and tumorigenesis. One of the key players regulating cell cycle progression is the F-box protein Skp2. Skp2 forms a SCF complex with Skp1, Cul-1, and Rbx1 to constitute E3 ligase through its F-box domain. Skp2 protein levels are regulated during the cell cycle, and recent studies reveal that Skp2 stability, subcellular localization, and activity are regulated by its phosphorylation. Overexpression of Skp2 is associated with a variety of human cancers, indicating that Skp2 may contribute to the development of human cancers. The notion is supported by various genetic mouse models that demonstrate an oncogenic activity of Skp2 and its requirement in cancer progression, suggesting that Skp2 may be a novel and attractive therapeutic target for cancers.

## INTRODUCTION

Skp2 (S-phase kinase–associated protein 2) belongs to the family of the F-box proteins. It was originally discovered by Beach and colleagues in 1995 because of its ability to interact with the cell cycle protein cyclin A[1]. Subsequent experiments revealed that Skp2 is involved in cell cycle progression[1]. Owing to its important role in regulating the stability of cell cycle inhibitors, such as p27, and ultimately affecting cell cycle progression, the research effort towards understanding Skp2 biological functions and its regulation is blooming and now under intensive study.

The Skp2 SCF complex consists of Skp1, Cul-1 (Cullin-1), F-box protein Skp2, and Rbx1 (also known as Roc1 and Hrt1). Skp2 contains the N-terminal domain, F-box domain, and C-terminal leucine-rich repeats (LRR)[2,3]. The crystal structure reveals that Skp2 interacts with Skp1 through its F-box domain, whereas it does not directly contact with Cul-1[4,5]. As a result, deletion of the Skp2 F-box domain prevents Skp2 from forming a Skp2 SCF complex, in turn compromising its SCF Skp2 E3 ligase activity. The N-terminal domain of Skp2 consists of the destruction domain (D-Box) that critically controls Skp2 stability, while Skp2 LRR are responsible for the interaction of Skp2 with its substrates. Unlike Skp1 and Cul-1, the Skp2 levels change during the cell cycle[6]. The Skp2 protein level is low in early G1 phase, while it is high during G1/S transition[6]. It is now known that this alteration in the Skp2 protein level during cell cycle progression is partly due to a change in its gene expression and protein stability.

*The first two authors contribute equally.
**Corresponding author.
©2010 with author.
Published by TheScientificWorld; www.thescientificworld.com

Post-translational modifications, such as phosphorylation, ubiquitination, sumoylation, and methylation, often regulate protein localization, stability, and activity. Although it has been known for a long time that Skp2 undergoes phosphorylation during cell cycle progression and growth factor stimulation[7], it remains unclear what kinases are involved and what role Skp2 phosphorylation plays. Recent studies reveal that Skp2 phosphorylation is triggered by Cdk2 and Akt kinases, which appear to play an important role in Skp2 stability, localization, and activity. In this review, we will summarize recent advances in the regulation of Skp2 activity and protein expression, with a particular emphasis on Skp2 phosphorylation and its potential implications in cancers.

## SKP2 BELONGS TO THE FAMILY OF F-BOX PROTEINS

There are 68 F-box proteins identified in the human genome, which are categorized into three classes based on the types of the substrate-interaction domains within them (Fig. 1)[2,8]. The first class of F-box proteins (FBXWs) includes those proteins containing the WD40 repeats, which are involved in protein-protein interaction. The best-known proteins in this class are β-TRCP and FBXW7, which are known to be involved in cell cycle regulation and tumorigenesis by targeting proteins involved in these processes. The second class of F-box proteins (FBXLs) includes those proteins containing the LRR. Skp2 (also known as FBXL1), is fitted into this category, and is well studied with several known protein substrates. The third class of F-box proteins (FBXOs) includes those proteins with other diverse domains in the C-terminal region. Although a large number of the F-box proteins are identified, only a few proteins, such as Skp2, β-TRCP, and FBXW7, have been well studied with characterized substrates.

## THE ROLE OF SKP2 IN PROTEIN UBIQUITINATION AND DEGRADATION

As mentioned above, Skp2 is a critical component of Skp2 SCF ubiquitin ligase, which is capable of inducing protein ubiquitination and subsequent proteasome-dependent degradation. The best-known Skp2 substrate is the p27 cell cycle inhibitor. Skp2 overexpression induces p27 ubiquitination and degradation, while Skp2 silencing reduces it. The binding of Skp2 to p27 requires its cofactor Cks1, as *Cks1* deficiency prevents Skp2 binding to p27, in turn leading to p27 up-regulation[9,10,11]. Similar to the phenotype observed in *Cks1*$^{-/-}$ MEFs, *Skp2*$^{-/-}$ MEFs display reduced cell proliferation, accompanied by enhanced p27 protein expression[12]. Interestingly, double deficiency for p27 and Skp2 rescues the cell proliferation defect in *Skp2*$^{-/-}$ MEFs[13], suggesting that p27 is a critical and relevant Skp2 substrate for Skp2 functions. The notion is supported by further *in vivo* evidence showing that double deficiency for *p27* and *Skp2* in mice rescues the reduced organ size and body weight observed in *Skp2*-deficient mice[13]. Importantly, Skp2 overexpression is found in various human cancer samples associated with poor prognosis and inversely correlated with p27 expression level[14,15,16,17,18,19]. These results suggest that p27 is a major physiological and pathological substrate for Skp2. In addition to p27, Skp2 also regulates ubiquitination and degradation of many other substrates (Table 1), although the physiological significance or relevance of these substrates for Skp2 functions remains to be determined. Among them, several cell cycle regulators (such as p21[20,21], p57[22], E2F-1[23], MEF[24], p130[25,26], Tob1[27], Cyclin D[21], Cyclin E[28], Smad4[29], Myc[30,31], B-Myb[32], and RASSF1A[33]), apoptosis regulators (such as Myc[30,31] and Foxo1[34]), DNA replication factors (such as Orc1p[35] and Cdt1[36,37]), DNA recombination factor Rag-2[38], DNA repair factor Brca2[39] and transcriptional elongation factor Cdk9[40], MKP1 involved in ERK signaling[41], and UBP43 involved in interferon signaling[42] are identified. These results suggest that apart from its known role in cell cycle progression and apoptosis, Skp2 may also participate in a wide range of biological processes. Future experiments using the genetic mouse models will be required to understand further whether those proteins are indeed relevant and important for Skp2 functions.

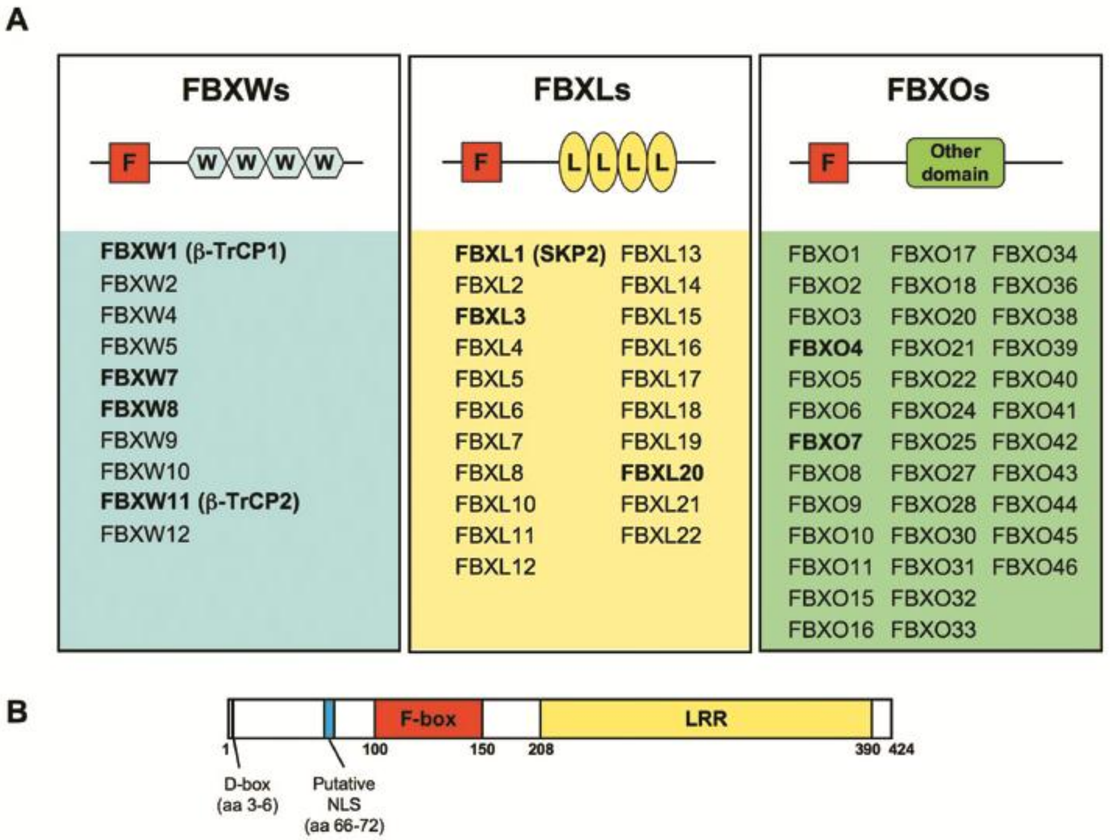

**FIGURE 1.** The family of human F-box proteins and domain structure of human Skp2 protein. (A) Based on the types of substrate-interaction domains, human F-box proteins are classified into three categories: FBXWs are those with WD40 repeats, FBXLs are those with LRR, and FBXOs are those with other diverse protein-interaction domains or no recognizable domains. Simplified conceptual schematic representations of these three classes are shown, including F-box motif (F), WD40 repeat (W), LRR (L), and other types of domains (other domain), and 68 human F-box proteins are classified and listed below, which have well-characterized or proposed substrates indicated in bold. (B) Functional domains of human Skp2 protein are indicated, including the destruction box (D-box), which is required for the APC/Cdh1-mediated degradation of Skp2 (amino acids 3–6), F-box motif, and LRR. A putative nuclear localization sequence (NLS) is also indicated (amino acids 66–72).

## THE ROLE OF SKP2 IN CELL SURVIVAL AND APOPTOSIS

Recent genetic and knockdown experiments reveal that in addition to its role in cell cycle progression, Skp2 also regulates cell survival and apoptosis. Knockdown of Skp2 by RNA interference induces apoptosis in various cell types[43,44,45]. Consistent with this observation, *Skp2*$^{-/-}$ MEFs also display a higher apoptosis rate compared with wild-type MEFs[12]. How does Skp2 regulate cell apoptosis? One possibility is that p27 accumulation in *Skp2* knockdown or null cells may contribute to cell apoptosis, as p27 overexpression induces cell cycle arrest and apoptosis. The pRB tumor suppressor was shown to interact with Skp2 and to prevent Skp2 from binding to p27, in turn inducing cell cycle arrest[46]. Interestingly, *pRB* knockdown was recently shown to overcome cell apoptosis induced by *Skp2* silencing[44], suggesting that activating the pRB-p27 pathway likely contributes to Skp2-mediated cell survival and apoptosis.

Another possibility that Skp2 regulates cell apoptosis may be through affecting p53 activity. One recent study showed that Skp2 does not affect p53 expression or interact with p53, but negatively regulates p53 acetylation and transcriptional activity by sequestering p300 from p53[43]. Skp2 knockdown induces and potentiates cell apoptosis upon stimulation with DNA damage agents in *p53*$^{+/+}$ HCT116 cells, but not in *p53*$^{-/-}$ HCT116 cells[43], suggesting that Skp2 likely regulates cell apoptosis in

**TABLE 1**
**The Known Skp2 Substrates and Their Functions***

| Substrate | Function | Regulation | Reference |
|---|---|---|---|
| p27 | Cell cycle inhibition | Skp2 induces the degradation of p27 and promotes cell cycle progression | 9-13 |
| p21 | Cell cycle inhibition | Skp2 induces the degradation of p21 and promotes cell cycle progression | 20,21 |
| p57 | Cell cycle inhibition | Skp2 induces the degradation of p57 and promotes cell cycle progression | 22 |
| E2F-1 | Cell cycle regulation/ Apoptosis | Skp2 induces the degradation of E2F-1 and downregulation of its transcriptional activity | 23 |
| MEF | Cell cycle regulation | Skp2 induces the degradation of MEF at the G1/S transition | 24 |
| p130 | Cell cycle inhibition | Skp2 induces the degradation of p130 and promotes G1/S transition | 25,26 |
| Tob1 | Cell cycle inhibition | Skp2 induces the degradation of Tob1 and promotes G1/S transition | 27 |
| Cyclin D | Cell cycle regulation | Skp2 induces the degradation of Cyclin D and regulates cell cycle progression | 21 |
| Cyclin E | Cell cycle regulation | Skp2 induces the degradation of Cyclin E and regulates cell cycle progression | 28 |
| Smad4 | Cell cycle arrest | Skp2 induces the degradation of cancer-derived Smad4 mutants | 29 |
| Myc | Cell cycle regulation/ Apoptosis | Skp2 triggers the degradation of Myc and enhances its transcriptional activity | 30,31 |
| Myb | Cell cycle regulation | Skp2 causes the degradation of Mycb, and Myb degradation may be required for controlling the cell cycle progression | 32 |
| RASSF1A | Cell cycle inhibition | Skp2 induces the degradation of RASSF1A and promotes G1/S transition | 33 |
| Foxo1 | Apoptosis | Skp2 triggers the degradation of Foxo1 and induces cell cycle arrest and apoptosis | 34 |
| Orc1p | DNA replication | Skp2 triggers the degradation of Orc1p to ensure that DNA replication is restricted to the S phase | 35 |
| Cdt1 | DNA replication | Skp2 triggers the degradation of Cdt1 at S phase to prevent re-replication | 36,37 |
| Rag-2 | DNA recombination | Skp2 triggers the degradation of Rag-2 and regulates V(D)J recombination | 38 |
| Brca2 | DNA repair | Skp2 triggers the degradation of Brca2 and increases cell proliferation potential | 39 |
| Cdk9 | Transcriptional elongation | Skp2 triggers the degradation of Cdk9 and regulates its transcriptional activity | 40 |
| MKP1 | ERK signaling | Skp2 triggers the degradation of MKP1 and provides the a positive feedback in ERK activation | 41 |
| UBP43 | Type 1 IFN signaling | Skp2 causes the degradation of UBP43 and may play a role in modifying type 1 IFN signaling | 42 |

* Skp2 interacts with and induces the ubiquitination and subsequent proteasome-dependent degradation of its substrates.

a p53-dependent manner. One caveat is that the efficiency of Skp2 knockdown may not reach to the threshold levels required for apoptosis. To address this possibility, it will be important to determine whether p53 inactivation rescues cell apoptosis observed in *Skp2*$^{-/-}$ MEFs.

Skp2 may also trigger Foxo1 degradation to regulate apoptosis. Foxo1 belongs to a family of Forkhead transcription factors, which induces cell cycle arrest and apoptosis [47,48]. Skp2 induces Foxo1 ubiquitination and degradation requiring Akt-mediated Foxo1 phosphorylation at Ser 256[34,47]. Skp2 overexpression attenuates Foxo1-mediated cell cycle arrest and apoptosis, and its expression is inversely correlated with Foxo1 expression in a mouse lymphoma model. However, it is unclear whether Foxo1 accumulation indeed contributes to the apoptotic phenotype observed in *Skp2*$^{-/-}$ MEFs.

## REGULATION OF SKP2 GENE EXPRESSION

Analyzing the Skp2 promoter reveals that several potential transcription factors, such as E2F1[49], NF-kB[50,51], SP1[52], CBF1[53], GABP (GA-binding protein)[54], and FoxM1[55], are involved. Indeed, CBF1, GABP, and FoxM1 are shown to bind to the Skp2 promoter region and are required for Skp2 gene expression (Fig. 2). Interestingly, GABP binding to the Skp2 promoter is found to be dependent on the cell cycle[54], supporting the idea that Skp2 gene expression is regulated by the cell cycle. Notch1 signaling also induces Skp2 gene expression by associating with CBF1, and triggers Skp2-dependent p21

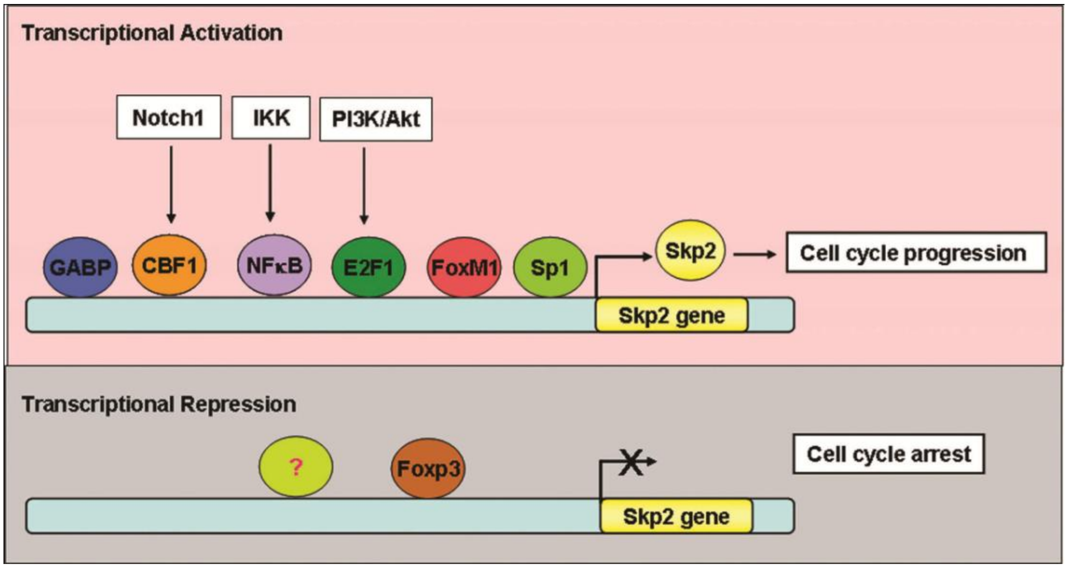

**FIGURE 2.** Regulation of Skp2 gene expression**.** GABP, controlled by cell cycle; CBF1, mediated by Notch signaling; NF-kB, triggered by IKK/NF-kB signaling; E2F1, regulated by PI3K/Akt signaling, are shown to bind to Skp2 promoter region and are required for Skp2 gene expression and cell cycle progression. FoxM1 and Sp1 have been also shown to regulate Skp2 transcriptional activation. Foxp3, a recent identified tumor suppressor, can bind to the Skp2 promoter and repress Skp2 gene expression to induce cell cycle arrest.

and p27 degradation and cell cycle progression[53]. Moreover, depletion of FoxM1 induces cell cycle arrest and polyploidy, similar to that observed in *Skp2* null cells[55], suggesting that FoxM1 may regulate cell progression and the genomic stability through the induction of Skp2 gene expression. IKK-NF-κB signaling known to be involved in inflammation and cancers also regulates Skp2 gene expression through the binding of p52/RelA or p52/RelB to the Skp2 promoter, in turn regulating p27 stability and cell cycle progression[50,51].

Skp2 gene expression is also regulated by phosphoinositol 3-kinase (PI3K)/Akt signaling as evidenced by the fact that inhibition of PI3K activity by LY294002 or Akt1 knockdown reduces Skp2 mRNA levels[56,57,58], although the underlying mechanism still remains elusive. One study suggests that Akt may trigger Skp2 gene expression by regulating E2F protein levels and its ability to bind to the Skp2 promoter[59]. However, it remains undetermined whether E2F is critical for PI3K/Akt-mediated Skp2 gene expression and protein expression. Several oncogenic signals, such as BCR-ABL and Her2/Neu, overexpressed in human cancers, are known to induce Skp2 gene expression through the PI3K/Akt signaling[60,61]. In a bone marrow transplantation leukemia animal model, *Skp2* deficiency is shown to delay BCR-ABL–induced leukemogenesis[62], suggesting that Skp2 up-regulation driven by BCR-ABL signaling plays an important role in leukemogenesis upon BCR-ABL overexpression.

Although several transcription factors responsible for Skp2 gene expression are identified, the transcriptional repressors for Skp2 are less clear. A recent report suggests that Foxp3 (Forkhead box p3), which is an X-linked breast cancer suppressor, is a Skp2 transcriptional repressor[63]. The earlier study suggests that Foxp3 is a transcriptional repressor for the Her2 oncogene, and mice heterozygous for Foxp3 developed a high rate of spontaneous breast cancer[64], suggesting that Foxp3 is a bona fide tumor suppressor. Foxp3 can also bind to the Skp2 promoter and represses Skp2 gene expression to induce cell cycle arrest. Interestingly, Skp2 overexpression is found in human breast cancer samples and is correlated with Foxp3 down-regulation[64], raising the possibility that Skp2 up-regulation may contribute to the development of breast cancer in *Foxp3*$^{+/-}$ mice. Future study by crossing *Skp2*$^{-/-}$ mice and *Foxp3*$^{+/-}$ mice is required to address this possibility.

## REGULATION OF SKP2 PROTEIN STABILITY

Skp2 protein stability is also regulated by the cell cycle. In addition to regulating its gene expression, Skp2 protein stability is regulated by various stimuli. Skp2 is a short-lived protein and its stability is regulated by a ubiquitination-dependent proteasome system. Skp2 ubiquitination is triggered by the E3 ubiquitin ligase APC (anaphase promoting complex)/Cdh1 complex in early G1 phase and results in Skp2 degradation[65,66] (Fig. 3). Silencing of Cdh1 leads to accumulation of Skp2 proteins, in turn promoting S-phase transition. This is in line with the fact that the Cdh1 protein level is low in G1/S transition, accompanied by a higher Skp2 expression[6,67]. The N-terminal D-box motif of Skp2 is responsible for Cdh1 binding, and removing this motif from Skp2 causes the resistance of Skp2 to Cdh1-mediated Skp2 ubiquitination and degradation[65,66].

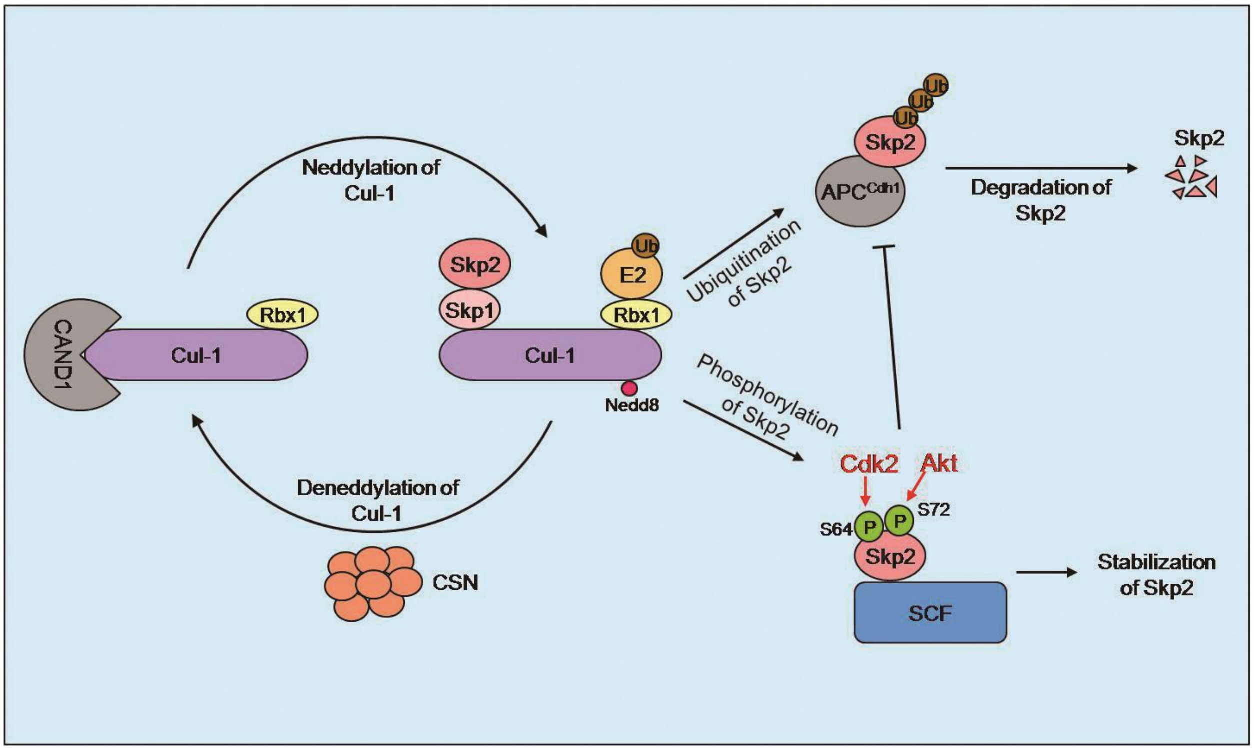

**FIGURE 3.** Regulation of Skp2 protein stability and the Skp2 SCF complex formation. The E3 ubiquitin ligase APC/Cdh1 complex ubiquitinates Skp2 through its binding to the D-box of Skp2 and leads to Skp2 degradation. Phosphorylation of Skp2 at Ser 64 and Ser 72 by Cdk2 and Akt, respectively, disrupts the interaction between Cdh1 and Skp2, thereby stabilizing Skp2. The formation of the Skp2 SCF complex can be regulated by neddylation and deneddylation cycles of Cul-1. The isopeptidase COP9 signalosome (CSN), consisting of an eight-subunit protein complex, induces deneddylation of Cul-1, which favors the interaction with CAND1, but prevents the binding of Cul-1 to Skp1 and Skp2. In contrast, neddylation of Cul-1 facilitates the assembly of the Skp2 SCF complex.

Recent biochemical studies reveal that Skp2 is phosphorylated by Akt and Cdk2 at residues Ser 64 and Ser 72[57,68], which are very close to the D-box motif within Skp2, and the phosphorylation of Skp2 on these residues prevents Cdh1 binding to Skp2, thereby attenuating Skp2 ubiquitination and degradation (Fig. 3). Accordingly, these results suggest that Akt not only regulates Skp2 gene expression through a mechanism that is currently not well understood, but also regulates Skp2 stability through promoting Skp2 phosphorylation. Since Akt and Cdk2 kinase activity is tightly regulated by the cell cycle, it remains to be determined whether the alteration in Akt and Cdk2 kinase activity indeed contributes to the dynamics of Skp2 protein expression observed in various phases of the cell cycle.

## REGULATION OF SKP2 SCF COMPLEX FORMATION AND ITS E3 LIGASE ACTIVITY BY SKP2 PHOSPHORYLATION

The integrity of Skp2 SCF complex formation is critical for Skp2 SCF E3 ligase activity. The post-translational modification such as neddylation regulates not only protein degradation, but also orchestrates protein-protein interaction[69]. It is proposed that neddylation of Cul-1 positively regulates Skp2 SCF complex formation and its E3 ligase activity. Neddylation of Cul-1 stabilizes the Skp2 SCF complex by preventing the binding of Cul-1 to Cand1, a negative regulator for the Skp2 SCF complex (Fig. 3). Cand1 preferentially interacts with unneddylated Cul-1 and prevents Cul-1 from binding to Skp1 and Skp2, in turn inactivating Skp2 SCF ligase activity *in vitro*[15,70,71]. However, it is unclear whether Cand1 also negatively regulates Skp2 SCF E3 ligase activity *in vivo*. A recent study reveals that although Cand1 competes the binding of Cul-1 to Skp1 and Skp2, the disruption of Cand1 and Cul-1 interaction paradoxically reduces Skp2 SCF E3 ligase activity[72], suggesting that the optimal interaction of Cul-1 with Cand1 is required for SCF E3 ligase activation. Future study by using the genetic mouse model will be required in order to understand the biological functions of Cand1 and its role in regulating the Skp2 SCF complex.

Neddylation of Cul-1 is promoted by the Skp2/Skp1 complex, which dissociates Cul-1 from Cand1[73], while deneddylation of Cul-1 is triggered by isopeptidase COP9/signosome (CSN) complex[74,75,76]. Although neddylation of Cul-1 appears to be important for the assembly of Skp2 SCF complex *in vitro*, the *in vivo* significance and relevance of this reaction in SCF E3 ligase activity remains to be determined.

The formation of the Skp2 SCF complex is also regulated by the PI3K/Akt signal and Cyclin D[77]. It is shown that activation of PI3K/Akt or overexpression of Cyclin D induces the formation of the Skp2 SCF complex, while silencing of Cyclin D or inhibition of the PI3K/Akt pathway by PTEN tumor suppressor or LY294002 reduces it[77]. Interestingly, the neddylation status of Cul-1 is reduced by inhibiting the PI3K/Akt pathway or silencing of Cyclin D, accompanied by the increase in the interaction of Cul-1 with Cand1[77]. However, the mechanism by which the PI3K/Akt signal regulates the neddylation of Cul-1 remains to be further determined.

In addition to Cul-1 neddylation, phosphorylation of Skp2 also regulates the formation of the Skp2 SCF complex. While Cdk2-mediated Skp2 phosphorylation at Ser 64 does not impact on the assembly of the Skp2 SCF complex, Akt-mediated Ser 72 phosphorylation of Skp2 positively regulates it[78]. We show that PI3K/Akt activity and Skp2 phosphorylation at Ser 72 are required for Skp2 SCF E3 ligase activity towards p27 ubiquitination[78]. How Skp2 phosphorylation at Ser 72 regulates Skp2 SCF complex and its E3 ligase activity is not yet clear. It is possible that Skp2 phosphorylation at Ser 72 by Akt may orchestrate Cul-1 neddylation and the interaction between Cul-1 and Cand1.

## SKP2 REGULATES CELL MIGRATION AND METASTASIS

Overexpression of Skp2 is frequently observed in numerous human cancers, including prostate cancer, which is inversely correlated with p27 expression[3,15]. These observations suggest that Skp2 may contribute to the development of human cancers. Indeed, accumulating evidence suggests that Skp2 displays a proto-oncogenic role *in vitro* and *in vivo*. For example, Skp2 is shown to cooperate with H-Ras$^{G12V}$ to induce cell transformation in soft agar assay and tumor formation assays in nude mice[79]. While in the transgenic mice model, Skp2 overexpression in the T-cell compartment by itself does not induce T-cell lymphomas; it cooperates with N-Ras to induce T-cell lymphomas with shorter latency and higher penetrance, resulting in a significant decrease in mice survival[80]. Moreover, prostate-specific overexpression of Skp2 in mice leads to prostate intraepithelial neoplasia[81], similar to the phenotypes observed in the transgenic mice with overexpression of constitutive active Akt1 in the prostates[82,83]. In line with these observations, we also show that Skp2 overexpression in prostate cancer cells markedly promotes prostate cancer cell growth and tumorigenesis in the xenograft tumor model[78], whereas overexpression of the Skp2 S72A mutant fails to do so[78]. Our study highlights the critical role of Skp2 phosphorylation at Ser 72 in Skp2 oncogenic activity.

Since Skp2 overexpression is also significantly associated with cancer metastasis[16,18,19,84,85], it raises the possibility that Skp2 may regulate cancer cell migration and metastasis. In support of this notion, we show that Skp2 *deficiency* displayed a defect in cell migration and metastasis, while Skp2 overexpression promoted cell migration and invasion[78,86]. Other studies also consistently show that Skp2 knockdown in cancer cells not only markedly reduces cell migration[87,88], but also inhibits tumor metastasis[89]. Importantly, we further show that RhoA is a critical downstream effector responsible for Skp2-mediated cell migration and metastasis[86]. Accordingly, these studies delineate the oncogenic roles of Skp2 in primary tumor formation, cell migration, and cancer metastasis.

## SKP2 PHOSPHORYLATION REGULATES SKP2 CYTOSOLIC LOCALIZATION AND FUNCTIONS

Skp2 is localized primarily in the nucleus in normal cells. However, Skp2 is relocalized to the cytoplasm during cancer progression in human cancers[16,18,19,85], although the underlying mechanism has not been clear until recently. Akt signaling appears to play an important role in Skp2 cytosolic localization (Fig. 4). We show that Akt phosphorylation at Ser 473 is significantly correlated with cytosolic Skp2 localization in human prostate and colon cancer samples[78], suggesting that Akt activation may contribute to the cytosolic Skp2 relocalization during the progression of human cancers. The notion is further supported by our recent report and others' demonstrating that Akt activation induced by growth factor IGF-1 (insulin-like growth factor-1) promotes Skp2 cytosolic localization, whereas inhibition of Akt activation prevents it[57,78,90].

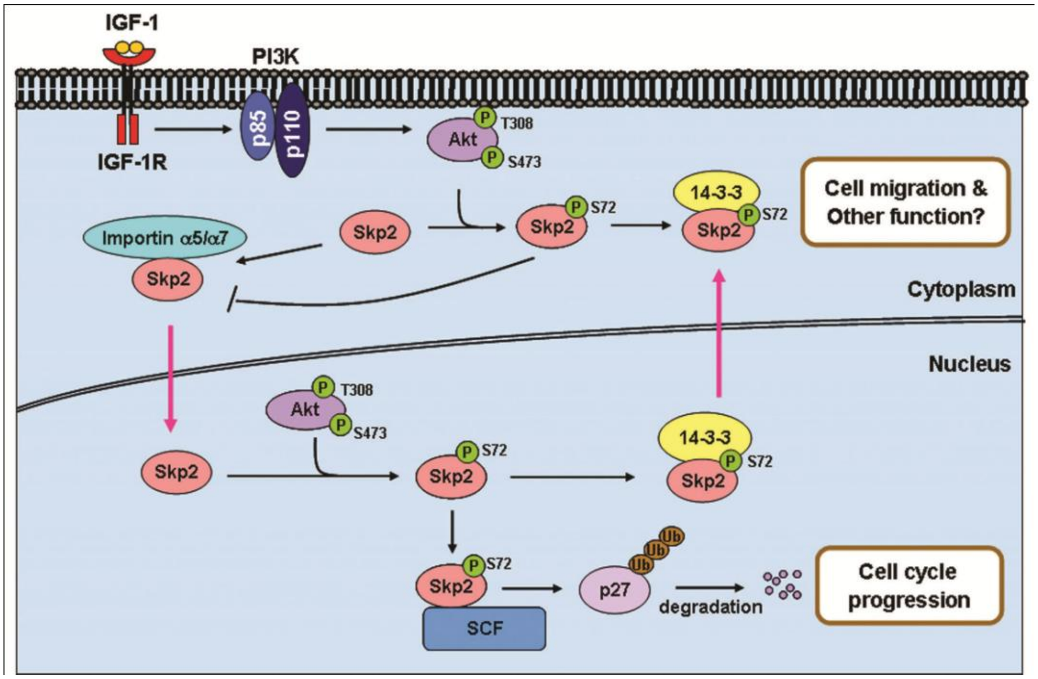

**FIGURE 4.** Akt-mediated phosphorylation of Skp2 regulates Skp2 cytosolic localization and functions. Upon IGF-1 stimulation, phosphorylation of nuclear Skp2 at Ser 72 by Akt promotes the interaction of Skp2 with 14-3-3, in turn facilitating Skp2 cytosolic localization. Phosphorylation of cytosolic Skp2 at Ser 72 prevents the interaction of Skp2 with importin α5 and α7, in turn preventing Skp2 nuclear import. One of the cytosolic Skp2 functions is to promote cell migration. Interestingly, phosphorylation of nuclear Skp2 at Ser 72 also enhances the Skp2 SCF complex formation and its E3 ligase activity, in turn promoting cell cycle progression and tumorigenesis.

To gain further insight into how Akt regulates Skp2 cytosolic localization, biochemical fractionation and immunofluorescence experiments reveal that Akt-mediated Skp2 phosphorylation at Ser 72 triggers Skp2 cytosolic localization, as the Skp2 S72D phosphomimetic mutant readily localizes in the cytoplasm, but the Skp2 phosphorylation dead mutant (Skp2 S72A) is resistant to Akt-mediated Skp2 cytosolic localization[57,78]. Notably, Akt-driven Skp2 cytosolic localization is inhibited by leptomycin B, a nuclear export inhibitor[78]. Accordingly, these results suggest that the mechanism by which Akt-mediated Skp2 cytosolic localization is due to the ability of Akt to induce Skp2 phosphorylation, in turn promoting Skp2 nuclear export.

Interestingly, the region where Skp2 phosphorylation occurs conforms to the 14-3-3 consensus-binding motif, indicating that Akt-mediated Skp2 phosphorylation at Ser 72 may facilitate the interaction between Skp2 and 14-3-3. 14-3-3 is an adaptor protein that interacts with target proteins to regulate their trafficking in a phosphorylation-dependent manner. Indeed, Akt is shown to promote the interaction between Skp2 and 14-3-3 dependently of Skp2 phosphorylation at Ser 72[57,78]. Silencing 14-3-3β expression inhibits the ability of Akt to promote Skp2 cytosolic localization[78], suggesting that Akt-mediated Skp2 phosphorylation at Ser 72 facilitates the interaction of Skp2 with 14-3-3, thereby relocalizing Skp2 from the nucleus to the cytoplasm.

Skp2 contains a putative nuclear localization signal (NLS) within the region where Skp2 phosphorylation by Akt takes place. Removing this putative NLS from Skp2 renders Skp2 to localize to the cytoplasm[57]. Skp2 is shown to interact with importin α5 and α7, which is known to import proteins containing the NLS into the nucleus, but not with importin α1[57]. Interestingly, Skp2 phosphorylation by Akt disrupts the interaction of Skp2 with importin α5 and α7[57], suggesting that another mechanism by which Skp2 phosphorylation induces Skp2 cytosolic localization is to prevent Skp2 nuclear import (Fig. 4).

What are the potential Skp2 functions in the cytoplasm? Although p27 is also relocalized to the cytoplasm upon Akt-mediated p27 phosphorylation at Ser 157, this p27 phosphorylation does not impact on its degradation[91,92,93,94]. Thus, it is very likely that Skp2 in the cytoplasm may not trigger p27 degradation. Consistent with this notion, we found that cytosolic Skp2 neither forms a complex with Cul-1 and Skp1 nor induces p27 degradation[78].

Interestingly, we found that cytosolic Skp2 rescues the cell migration defect in *Skp2*$^{-/-}$ MEFs[78], suggesting that cytosolic Skp2 regulates cell migration independently of its ability to regulate p27 ubiquitination and degradation. Accordingly, our study suggests that the PI3K/Akt signal and Skp2 phosphorylation at Ser 72 may serve as molecular switches to orchestrate Skp2 cytosolic localization during cancer progression, in turn regulating the role of Skp2 in cancer cell invasion and metastasis.

This exciting finding opens up a new avenue for Akt and Skp2 research, and likely provides a novel paradigm for cancer treatment. Future experiments addressing how Skp2 regulates cell migration will yield further insight into how Skp2 may regulate cellular functions independently of its ability to regulate p27 degradation.

## THERAPEUTIC IMPLICATIONS

The 15-year research about the Skp2 signaling has yielded several exciting and novel discoveries, and suggests that Skp2 targeting may be a very attractive approach to treat human cancers. Three recent reports using the genetic approaches provide the compelling evidence demonstrating that Skp2 is required for tumorigenesis upon BCR-ABL overexpression, Pten loss, or pRB inactivation[44,62,95]. Given that Skp2 overexpression is shown to induce or potentiate cancer development in mouse models and that its overexpression is observed in a variety of human cancer specimens, it is possible that Skp2 targeting can be very effective for many types of human cancers. Future experiments using the genetic approaches by crossing *Skp2* null mice with other tumor mouse models will be required in order to determine whether Skp2 is also required for cancer maintenance in various cancers.

Despite the important roles of Skp2 in cell proliferation, survival, and cancer development, specific Skp2 inhibitors have not yet been identified. However, two small molecules targeting other components of the Skp2 SCF complex were recently identified and proven to be promising for treating human cancers. One study showed that a small molecule targeting Skp2 SCF E3 ligase activity towards p27 ubiquitination causes cell arrest, apoptosis, and autophagy in leukemia cells[96]. Another study identified a small molecule inhibitor (MLN4924), which targets Nedd8-activating enzyme, thereby affecting Cul-1 neddylation and Skp2 SCF complex formation[97]. MLN4924 reduced Cul-1 neddylation, accompanied by inducing p27 accumulation, cell arrest, apoptosis, and senescence[95,97]. In xenograft tumor models, MLN4924 was shown to exhibit potent effects on suppressing tumor growth *in vivo*[95,97].

Accordingly, these studies provide the convincing proof of principle evidence that targeting the Skp2 SCF complex can be an effective strategy for cancer treatment. Thus, identification of Skp2 inhibitors will be urgently needed in the near future and may be beneficial for patients with various types of cancers.

## CONCLUSION AND FUTURE DIRECTIONS

Akt signaling plays a crucial role in a myriad of biological functions, such as cell proliferation, survival, migration, metabolism, and tumorigenesis[98,99,100,101,102,103]. These Akt functions are achieved primarily through the phosphorylation of multiple Akt downstream effectors by Akt. Interestingly, Skp2 is also shown to play overlapped functions as Akt does, suggesting that Skp2 may cooperate with Akt signaling to regulate these biological functions. Indeed, recent studies suggest that Skp2 is a novel substrate of Akt, and Skp2 phosphorylation by Akt regulates Skp2 stability, activity, and subcellular localization, in turn promoting Skp2-mediated cell cycle progression, cell migration, and tumorigenesis. These findings have advanced our current understanding of how Skp2 signaling is regulated and suggest that Skp2 may be an important downstream effector mediating numerous biological functions of Akt.

Many types of human cancers display Skp2 overexpression. Recent studies demonstrate that Skp2 overexpression promotes cancer progression and metastasis, while its deficiency inhibits these processes, suggesting that targeting Skp2 may be an ideal strategy for human cancer treatment. These studies therefore call for an urgent need to design small molecule inhibitors of Skp2 for target human cancers. Alternatively, given that Skp2 activity is regulated by numerous mechanisms such as affecting its gene expression, protein stability, and Skp2 SCF complex formation, targeting these mechanisms can be also considered for potential strategies for human cancers. In the case of the regulation of Skp2 gene expression, the inhibition of Notch, IKK/NF-κB, or Akt signaling is expected to shut down Skp2 gene expression. Indeed, small molecules targeting these pathways have already been developed and tested in clinical trials. In terms of the control of Skp2 protein stability, small molecule inhibitors targeting Akt and CDK2 activity, which are shown to stabilize Skp2 protein stability, are expected to trigger Skp2 rapid degradation, in turn inhibiting cancer development. Finally, in terms of the regulation of the Skp2 SCF complex formation, targeting Cul-1 neddylation is proven to be a good way to disrupt the Skp2 SCF complex formation. Supporting such a notion came from a recent success of using MLN4924 in preclinical mouse tumor models, which is known to disrupt the Cul-1 neddylation and Skp2 SCF complex formation.

Several important questions remain mysterious and warrant future investigations. What functions do other known Skp2 substrates play in Skp2-mediated cell proliferation, apoptosis, and tumorigenesis? What exact roles does Cand1 play in Skp2 SCF complex formation and cancer progression? How does Skp2 phosphorylation regulate cell migration? Is Skp2 globally involved in cancer development in various human tissues? Addressing these important questions will lead to comprehensive understanding of how the Skp2 SCF complex is regulated and its roles in cancer progression and metastasis.

## ACKNOWLEDGMENTS

We thank the members of Dr. Lin's lab for their critical reading and comments on our manuscript. This work is supported by the M.D. Anderson Research Trust Scholar Fund and New Investigator Award from the Department of Defense (PC081292) to H.K. Lin.
